# Dual Regulation of Corneodesmosome Formation by Shotokuseki Extract Enhances Skin Barrier Homeostasis

**DOI:** 10.3390/molecules30234592

**Published:** 2025-11-29

**Authors:** Kei Tsukui, Masamitsu Suzuki, Miyu Amma, Shin-ichi Kawaguchi, Yoshihiro Tokudome

**Affiliations:** 1 The United Graduate School of Agricultural Sciences, Kagoshima University, Kagoshima 890-0065, Japan; 2 Zeria Pharmaceutical Co., Ltd., Tokyo 103-8351, Japan; 3 Center for Bioresource Education and Research, Saga University, Saga 847-0021, Japan; 4 Graduate School of Advanced Health Sciences, Saga University, Saga 840-8502, Japan; 5 Graduate School of Science and Engineering, Saga University, Saga 840-8502, Japan; 6 Laboratory of Cosmetic Sciences, Institute of Ocean Energy, Saga University, Saga 840-8502, Japan

**Keywords:** Shotokuseki extract, stratum corneum, corneodesmosome, ion

## Abstract

Corneodesmosomes are specialized intercellular junctions that mediate adhesion between corneocytes in the stratum corneum (SC). The degradation of these structures is regulated by kallikrein-related peptidases (KLKs) and their inhibitors. This study aimed to elucidate the effects of Shotokuseki extract (SE), a substance rich in various trace elements, on molecules related to SC adhesion using a three-dimensional cultured human epidermis model. SE was applied to a three-dimensional epidermis model, and analyses were conducted on gene expression, protease activity, protein levels, and tissue structure. SE treatment significantly upregulated the mRNA expression of corneodesmosomal components (desmoglein1, desmocollin1, and corneodesmosin) and the major protease inhibitor serine peptidase inhibitor Kazal type 5. Concurrently, SE increased the mRNA expression of the trypsin-like protease KLK5,while significantly decreasing the mRNA expression and activity of the chymotrypsin-like protease KLK7. Although no significant changes were observed in the protein levels of corneodesmosomal components, histological analysis revealed that SE significantly increased the ratio of SC thickness to total epidermal thickness. These findings suggest that SE contributes to the homeostasis of the SC by simultaneously promoting the expression of genes encoding corneodesmosomal components, and regulating the balance of the protease/inhibitor system involved in their degradation. The selective suppression of KLK7 activity may appropriately regulate the final stage of desquamation, thereby stabilizing barrier function.

## 1. Introduction

The barrier function of the stratum corneum, the outermost layer of the skin, plays a pivotal role in protecting the body against physical and chemical damage, and its moisturizing function prevents transepidermal water loss [1]. Its structure is considered similar to a brick-and-mortar wall, with corneocytes as the bricks and intercellular lipids as the mortar. The maintenance of this layered architecture relies on physical support provided by intercellular lipids and specialized intercellular adhesion structures termed corneodesmosomes [2,3].

The desquamation of keratinocytes from the outermost layer of the skin is regulated by the synthesis and degradation of corneodesmosomes, which are composed of desmoglein 1 (DSG1), desmocollin 1 (DSC1), and corneodesmosin (CDSN). A group of serine proteases known as kallikrein-related peptidases (KLKs) play a major role in the degradation of these structures. Within the KLK family, KLK5 functions as the main enzyme that degrades DSG1, DSC1, and CDSN, while KLK7 contributes to the degradation of DSC1 and CDSN. KLK5 has trypsin-like activity, while KLK7 has chymotrypsin-like activity. These enzymes promote the desquamation of the stratum corneum [4,5,6,7]. Abnormal increases in protease activity can lead to excessive degradation of corneodesmosomes and impaired skin barrier function. Indeed, KLK dysfunction has been reported to play a significant role in the pathogenesis of various inflammatory skin diseases, including Netherton syndrome, atopic dermatitis (AD), psoriasis, and rosacea [8,9,10,11,12,13]. In healthy skin, serine protease activity is carefully controlled by endogenous protease inhibitors. Some of the main inhibitors are the lymphoepithelial Kazal-type related inhibitor (LEKTI), which is encoded by the SPINK5 gene; the secretory leukocyte protease inhibitor (SLPI); and elafin, which is encoded by peptidase inhibitor 3 (PI3). LEKTI is a major KLK5 inhibitor, and functional variants of the SPINK5 gene cause Netherton syndrome, which is characterized by three main symptoms: ichthyosis, hair abnormalities, and an atopic constitution. Conversely, SLPI and elafin primarily inhibit neutrophil-derived elastase. They are expressed at low levels in normal skin, but their expression increases in inflammatory environments, such as those associated with psoriasis. They are also believed to play a role in tissue protection [14,15].

In the epidermis, the extracellular calcium concentration gradient functions as a primary trigger for keratinocyte differentiation and the maintenance of barrier function homeostasis. Disruption of this gradient inhibits keratinocyte differentiation and barrier formation [16]. Extracellular calcium signaling is transmitted via calcium-sensing receptors (CaSRs), which are essential for calcium-dependent differentiation responses in keratinocytes [17]. In addition to calcium, transition trace elements (particularly zinc) regulate keratinocyte functions through strict transporter control [18]. For example, the expression of zinc transporter 2 is increased during differentiation and is functionally associated with the induction of differentiation marker expression [19]. Furthermore, basic and clinical research have demonstrated that other trace elements, including cerium, copper, selenium, and iron, are also involved in skin physiology and pathology [20,21]. However, their role at the molecular level within the multilayered epidermis has not been fully investigated yet.

Shotokuseki extract (SE) is a water-soluble component extracted from Shotokuseki shale that contains various trace elements. We previously reported the ion concentrations in SE measured by atomic absorption spectrometry and inductively coupled plasma-mass spectrometry [22,23]. The analysis revealed that SE contains Na, Mg, Al, K, Ca, Fe, Zn, and Ce at concentrations of 0.03, 0.14, 4.45, 0.01, 0.21, 4.48, 0.004, and 0.0004 mM, respectively. Previous studies have reported that SE affects keratinocyte differentiation and calcium signaling. Additionally, SE has been shown to promote the production of natural moisturizing factors and intercellular lipids in the stratum corneum of three-dimensional cultured epidermal cells [22,23,24]. However, the specific effects of SE on the synthesis of corneodesmosomal constituent molecules that contribute to the adhesion and detachment of the stratum corneum and its influence on the expression of serine proteases and protease inhibitors that regulate their degradation remain unclear. SE has been used as an ingredient in cosmetic products, such as lotions and creams, for more than 40 years. However, despite its long history of use, the effects of SE on the skin and its underlying mechanisms have not been investigated in detail. SE contains many inorganic ions, some of which, such as calcium and magnesium, have been implicated in epidermal differentiation and barrier formation. However, SE also includes several trace elements whose biological roles remain unclear. To distinguish the effects of major biologically relevant ions from those attributable to other minor components, we compared SE with an ion mixture (IM) formulated to match the concentrations of the key ions present in SE. This approach enables the identification of SE-specific activities that cannot be explained solely by its major ionic constituents.

This study was established to clarify the effects of SE on intercellular adhesion in the stratum corneum of three-dimensional cultured epidermis. Specifically, we focus on controlling the expression of corneodesmosomes, and serine proteases and their inhibitors, and evaluate the effects of SE on these molecules. We also examine whether the composition and balance of ions contained in SE contribute to maintaining the structural homeostasis of the stratum corneum.

## 2. Results

### 2.1. Effects of Expression of Genes Encoding Corneodesmosomal Components, Serine Proteases, and Serine Protease Inhibitors

**Figure 1:**
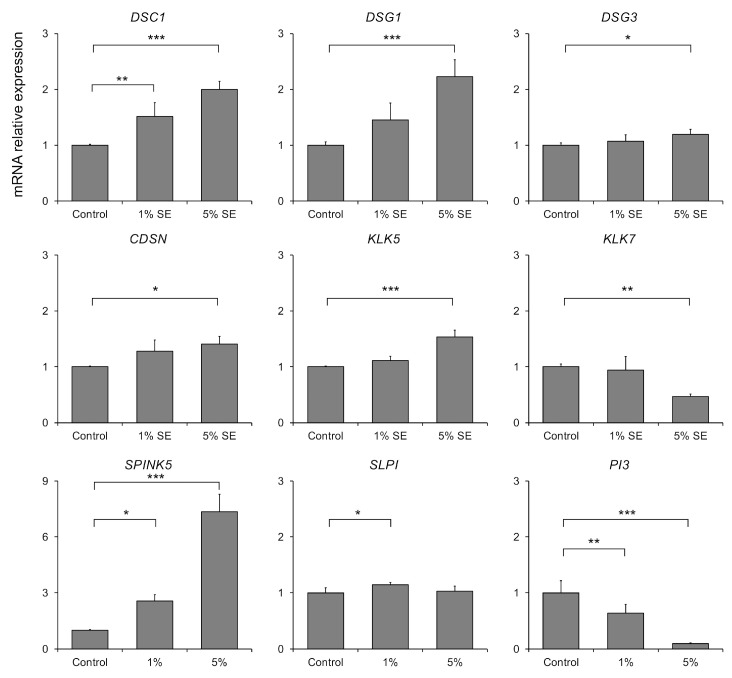
Effects of expression of genes encoding corneodesmosomal components, serine proteases, and serine protease inhibitors in three-dimensional human epidermis. Data are expressed as mean ± S.D. of four independent experiments. Statistical significance was evaluated using Dunnett’s test (* *p* < 0.05, ** *p* < 0.01, *** *p* < 0.001).

The effects of SE on the expression of mRNA for encoding corneodesmosomal components, serine proteases, and serine protease inhibitors in a three-dimensional epidermis model were investigated. The expression of the corneodesmosomal constituents DSC1, DSG1, and CDSN was significantly increased by 5% SE treatment. Similarly, the expression of the serine protease KLK5 mRNA was significantly increased by 5% SE treatment, whereas the expression of KLK7 mRNA was significantly decreased. Furthermore, SE treatment significantly increased the mRNA expression of serine protease inhibitors SPINK5 and SLPI, and significantly decreased the mRNA expression of PI3 (Figure 1).

### 2.2. Effects of Serine Protease Activities

**Figure 2:**
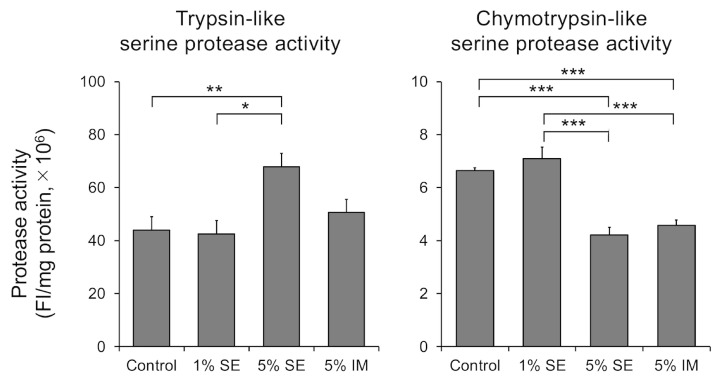
Effects of serine protease activities in three-dimensional human epidermis. Data are expressed as mean ± S.D. of four independent experiments. Statistical significance was evaluated using Tukey’s post hoc multiple comparison test (* *p* < 0.05, ** *p* < 0.01, *** *p* < 0.001).

The activity of trypsin- and chymotrypsin-like serine proteases in the three-dimensional epidermis treated with SE or IM was evaluated. Trypsin-like serine protease activity significantly increased with 5% SE, while chymotrypsin-like serine protease activity significantly decreased with both 5% SE and 5% IM (Figure 2).

### 2.3. Effects on Protein Expression of Corneodesmosomal Components

**Figure 3:**
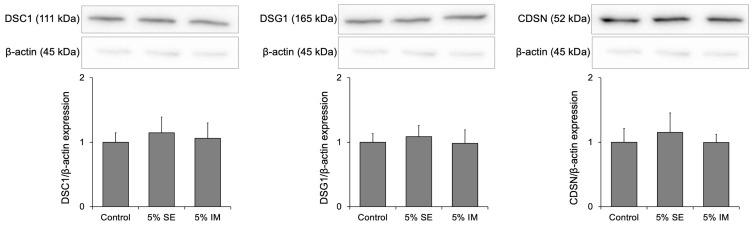
Effects on protein expression of corneodesmosomal components in three-dimensional human epidermis. Data are expressed as mean ± S.D. of four independent experiments. Statistical significance was evaluated using Tukey’s post hoc multiple comparison test.

The effects of SE and IM treatment on the expression of desmosome proteins in three-dimensional epidermis were analyzed. The expression of the desmosomal proteins DSC1, DSG1, and CDSN did not change with SE and IM treatment (Figure 3).

### 2.4. Effects of Epidermal Morphology

The structure of the three-dimensional human epidermis after treatment with SE or IM was observed using various microscopes.

**Figure 4:**
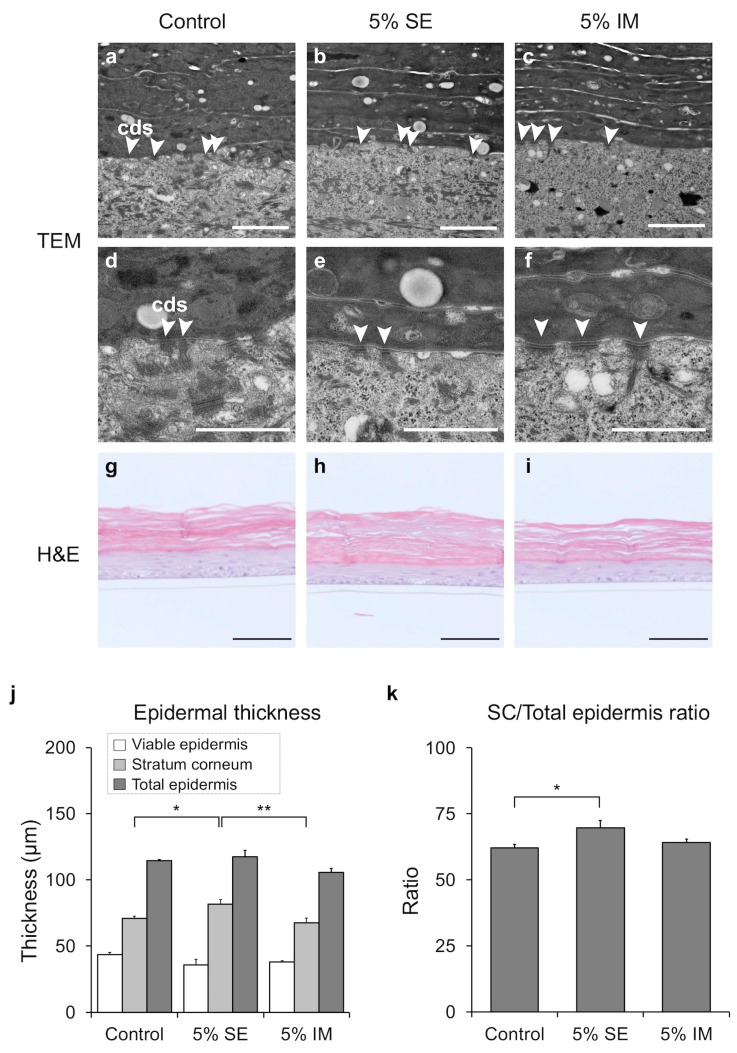
Effects of epidermal morphology in three-dimensional human epidermis. (**a**–**f**) Representative TEM images of ultrafine structures. The white arrow indicates corneodesmosomes (cds). Scale bars: 2 µm (**a**–**c**) and 1 µm (**d**–**f**). (**g**–**i**) Representative H&E images. Scale bars: 100 µm. (**j**) Results of analysis of epidermal thickness. (**k**) SC/total epidermal thickness ratio. Data are expressed as mean ± S.D. of three independent experiments. Statistical significance was evaluated using Dunnett’s test (* *p* < 0.05, ** *p* < 0.01).

TEM observation confirmed the presence of corneodesmosomes at the boundary between the stratum corneum and the granular layer (Figure 4a–f). Furthermore, the granular layer was found to contain keratohyalin granules, lamellar bodies, and lipid droplets, and to have a structure similar to that of the epidermis in vivo.

Next, H&E staining demonstrated that the epidermis was exhibited an ordered structure, and acellular keratinocyte formation was observed within the stratum corneum (Figure 4g–i). A significant increase in stratum corneum thickness observed with 5% SE treatment (Figure 4j). In addition, the stratum corneum (SC)/total epidermis ratio significantly increased (Figure 4k).

## 3. Discussion

This study investigated the effects of SE containing various trace elements on intercellular adhesion-related molecules in the stratum corneum of a three-dimensional cultured human epidermis model. The results showed that SE regulates stratum corneum turnover by controlling the expression of proteases and protease inhibitors. These proteases and inhibitors regulate the synthesis and degradation of the stratum corneum’s adhesive structure, which potentially explains the mechanism underlying this effect.

One of the most important findings of this study was that SE significantly increased the mRNA expression of DSG1, DSC1, and CDSN, which are major components of corneodesmosomes (Figure 1). These are essential for cell adhesion, which is responsible for the physical strength of the stratum corneum, and their increased expression suggests the formation of a stronger stratum corneum barrier. Interestingly, SE also increased the mRNA expression of KLK5, a major trypsin-like protease that degrades these adhesive molecules. At first glance, this seems contradictory, as SE promotes both the formation and the degradation of adhesive structures. However, SE also significantly increased the expression of SPINK5, a gene that encodes LEKTI, a major endogenous inhibitor of KLK5.

The balance between the serine protease KLK5 and its endogenous inhibitor SPINK5 plays a central role in maintaining the stability of the epidermal barrier. An imbalance in the regulation of this balance causes the excessive proteolysis of keratin desmosome proteins, leading to barrier dysfunction. This is evident from the fact that loss-of-function mutations in the SPINK5 gene cause Netherton syndrome, which is characterized by severe barrier dysfunction. Furthermore, the selective regulatory effect of SE on protease activity strongly supports this conclusion. The dual effect of “increased trypsin-like activity” and “decreased chymotrypsin-like activity” is completely consistent with the effects reported by Kobashi et al. following stimulation with a high concentration of calcium [25]. Our results suggest that SE helps regulate this balance, thereby contributing to the resilience of the desmoglein-mediated cell–cell junctions and the maintenance of homeostasis throughout the stratum corneum (Figure 1 and Figure 2).

This similarity strongly supports the hypothesis that SE primarily regulates protease activity by activating physiological control pathways involving calcium ions. It is thought that KLK5 activates the degradation cascade and that KLK7 promotes subsequent degradation. Selective inhibition of KLK7 activity is believed to effectively regulate the final stage of stratum corneum desquamation, thereby stabilizing barrier function. The direct or indirect inhibition of KLK7 activity by SE may appropriately delay the final stage of stratum corneum desquamation and prevent immature keratinocytes from desquamating. As a result, SE is believed to improve the adhesive strength of the stratum corneum. It also contributes to the stabilization of the barrier function. Furthermore, a decrease in chymotrypsin-like activity was observed with IM. This suggests that the ions contained in SE and IM, particularly calcium and zinc, which function as cofactors and allosteric regulators of enzymes, may be involved in this effect [26].

Histological observations indicate that SE promotes the differentiation of keratinocytes, thereby promoting the formation of the stratum corneum structure (Figure 4j,k). This differentiation-promoting effect of SE supports the findings in our previous reports [22,24]. The three-dimensional cultured epidermis used in this experiment differs from actual living epidermis in that it does not undergo desquamation of the stratum corneum [27]. Therefore, it is necessary to examine the effect of SE accumulation on the elimination of keratinocytes.

This study revealed a significant increase in the mRNA expression of DSC1, DSG1, and CDSN. Although we did not observe significant changes in the protein levels of corneodesmosomal components (Figure 3), this may be related to the timing of sample collection or the inherent turnover and degradation rates of these proteins. Corneodesmosomal proteins are subject to regulated proteolysis during epidermal differentiation, and transient changes in protein levels may have occurred outside the sampling window. Future studies with more frequent time points or complementary assays assessing protein turnover may provide further insights into the dynamic regulation of these proteins by SE.

In future studies, there will be a need for further detailed analyses to elucidate the mechanisms of SE’s effects on skin barrier function, particularly regarding protein localization and post-translational modification. Meanwhile, Kobashi et al. suggested that calcium plays a major role in regulating KLK activity. However, comparative analysis using IM suggested the presence of other trace elements contained in SE and their interactions. Further clarification of the effects of ions specific to SE is necessary.

This study demonstrated that SE promotes the expression of genes encoding components of corneodesmosomes while also regulating the balance of the protease and protease inhibitor systems that are involved in their degradation and control. This suggests that SE could potentially control the homeostasis of the stratum corneum. This mechanism of action, which controls turnover, provides scientific evidence that SE can be an effective skincare ingredient for dry and sensitive skin with impaired barrier function. It is anticipated that future work will elucidate the detailed mechanism involved in using disease models and research to identify the roles of different types of ions.

SE has been used in cosmetic formulations for over 40 years. Despite this long history of use, the mechanisms underlying its skin-beneficial effects have remained largely unexplored. Our findings suggest that SE exerts functional effects beyond simple moisturization, potentially by modulating the epidermal ion balance. There is precedent for mineral-rich water being used in skincare; for example, thermal spring water has been reported to reduce TEWL and improve skin hydration when incorporated into a cosmetic gel formulation. Such evidence underscores the potential of inorganic ions in topical applications. Taken together, SE with its complex and physiologically relevant ion composition, may represent a promising candidate for the development of next-generation functional skincare products

## 4. Materials and Methods

### 4.1. Materials

SE was provided by IONA International Corporation (Tokyo, Japan), a member of the Zeria Group (Tokyo, Japan). LabCyte EPI-MODEL 6D (three-dimensional human epidermis model) and culture medium were purchased from Japan Tissue Engineering (Gamagori, Aichi, Japan). Primers and Qubit^®^ RNA Broad Range Assay Kits and anti-DSG1 antibody (Cat# 32-6000) were purchased from Thermo Fisher Scientific (Waltham, MA, USA). RNAiso Plus, PrimeScript™ RT Reagent Kit, and TB Green™ Premix ExTaq were purchased from Takara Bio Inc. (Kusatsu, Shiga, Japan). Fluorescently labeled peptides were purchased from Peptide Institute, Inc. (Osaka, Japan). Anti-β-actin antibody (Cat# 4970) was purchased from Cell Signaling (Danvers, MA, USA). Anti-DSC1 antibody (Cat# 88099) was purchased from Novus Biochemicals (Centennial, CO, USA). Anti-CDSN antibody (Cat# 13184-1-AP) was purchased from Proteintech Group, Inc. (Chicago, IL, USA). Polyclonal Goat Anti-Rabbit Immunoglobulins/HRP was purchased from Agilent Technologies (Santa Clara, CA, USA). Polyvinylidene difluoride (PVDF) membranes and Western blotting detection reagents were purchased from Cytiva (Tokyo, Japan). Quetol-812 was purchased from Nisshin EM Co. (Tokyo, Japan). All other chemicals and solvents were purchased from Fujifilm Wako Pure Chemical Corporation (Osaka, Japan).

### 4.2. Preparation of SE and IM Solutions

The SE used in this study was provided by IONA International Corporation, a member of the Zeria Group, and manufactured following their proprietary and standardized procedures. Purified water was added to Shotokuseki, a type of shale, and the mixture was allowed to stand before the minerals were removed, yielding a solution equivalent to 2% (*w*/*v*) Shotokuseki. The IM solution was prepared in accordance with a previously described method [23]. This solution contained 0.03 mM Na, 0.01 mM K, 0.14 mM Mg, 0.21 mM Ca, and 0.004 mM Zn, corresponding to the concentrations of these ions in SE. These five ions were selected for comparison with other trace elements such as Al and Fe. They are considered major biologically relevant ions, and numerous studies have reported their functional roles in the epidermis. This ensured that SE and IM were compared under equivalent ion concentration conditions.

### 4.3. Cell Culture

In accordance with the manufacturer’s protocol, the three-dimensional human epidermis model was pre-incubated for 3 h prior to the daily topical application of SE or IM solution from the stratum corneum side, over 2–8 days. SE was applied at 1% or 5% and IM was applied at 5% to the three-dimensional human epidermis. The three-dimensional human epidermis was maintained in the manufacturer’s proprietary culture medium, which was replenished daily. Incubation was carried out at 37 °C in a humidified atmosphere containing 5% CO_2_.

### 4.4. RNA Extraction and Reverse-Transcription Quantitative PCR

After applying SE to the three-dimensional human epidermis for 24 h, total RNA was isolated from three-dimensional human epidermis using RNAiso Plus, following the manufacturer’s protocol. RNA concentration was determined using a Qubit^®^ 3.0 Fluorometer (Thermo Fisher Scientific) with the Qubit^®^ RNA Broad Range Assay Kit. cDNA was synthesized using the PrimeScript™ RT Reagent Kit on a GeneAmp^®^ PCR System 9700 thermal cycler (Thermo Fisher Scientific).

Quantitative PCR was conducted using the LightCycler^®^ 96 System (Roche, Basel, Switzerland) with TB Green^®^ Premix Ex Taq™. The amplification protocol consisted of initial denaturation at 95 °C for 30 s, followed by 45 cycles of 95 °C for 5 s and 60 °C for 30 s. Relative gene expression levels were calculated using the ΔΔCt method. Primer sequences are listed in Supplementary Table S1. mRNA expression levels in untreated control samples were set to 1.0, and expression in treated samples was expressed relative to this value.

### 4.5. Protease Activity Assay

After applying SE or IM to the three-dimensional human epidermis for 8 days, protease activity in the lysates of the three-dimensional human epidermis was assessed using fluorogenic peptide substrates. Briefly, epidermal lysate was mixed with 100 µL of either a trypsin-like serine protease-specific substrate (Boc–Phe–Ser–Arg–7-amino-4-methylcoumarin [MCA]) or a chymotrypsin-like serine protease-specific substrate (Phe–MCA) in 10 mmol/L Tris-HCl buffer (pH 7.8). The mixture was incubated at 37 °C for 24 h. Enzymatic activity was quantified by measuring fluorescence intensity (excitation/emission: 380/460 nm) using a microplate reader (SpectraMax iD5; Molecular Devices, San Jose, CA, USA).

### 4.6. Western Blotting

After applying SE or IM to the three-dimensional human epidermis for 48 h, total protein was extracted from the three-dimensional human epidermis using radioimmunoprecipitation assay buffer. Protein concentrations were determined using the bicinchoninic acid assay. Samples were mixed with loading buffer (0.1 M Tris-HCl, pH 6.8, 25% glycerol, 5% SDS, 0.01% bromophenol blue, and 5% 2-mercaptoethanol) and denatured by heating at 95 °C for 3 min. Proteins were separated by 10% sodium dodecyl sulfate-polyacrylamide gel electrophoresis (SDS-PAGE), followed by transfer to PVDF membranes. The membranes were blocked in Tris-buffered saline containing 0.1% Tween^®^ 20 (TBS-T) and 5% skim milk for 1 h at room temperature. Subsequently, the membranes were incubated with primary antibodies diluted in 5% skim milk either for 1 h at room temperature or overnight at 4 °C. The following primary antibodies were used: anti-DSC1 (300:1), anti-DSG1 (500:1), anti-CDSN (1000:1), and anti-β-actin (1000:1). After washing, the membranes were incubated with horseradish peroxidase (HRP)-conjugated secondary antibodies for 1 h at room temperature. Protein bands were detected using the Amersham™ ECL Prime Western Blotting Detection Reagent and visualized with a LAS-3000 imaging system (FUJIFILM, Tokyo, Japan). Densitometric analysis was performed using ImageJ software version 1.53a (NIH, Bethesda, MD, USA).

### 4.7. Transmission Electron Microscopy (TEM) Sample Preparation

After applying SE or IM to the three-dimensional human epidermis for 8 days, the tissue was fixed with 2% paraformaldehyde and 2% glutaraldehyde in 0.1 M cacodylate buffer (pH 7.4) at 4 °C overnight. After fixation, the samples were washed three times with 0.1 M cacodylate buffer for 30 min each at 4 °C, followed by post-fixation with 2% osmium tetroxide in 0.1 M cacodylate buffer at 4 °C for 2 h. The samples were then dehydrated through a graded ethanol series. For resin infiltration, the samples were treated with propylene oxide (PO) at room temperature for 30 min, and then incubated in a 1:1 mixture of PO and Quetol-812 (Nisshin EM Co., Tokyo, Japan) at room temperature for 1 h. Samples were embedded in Quetol-812 and polymerized at 60 °C for 48 h. Ultrathin sections (70 nm thick) were cut using an ultramicrotome (Ultracut UCT; Leica, Vienna, Austria) equipped with a diamond knife and collected on copper grids. The sections were stained with 2% uranyl acetate for 15 min and lead stain solution for 3 min at room temperature. Transmission electron microscopy (TEM) was performed using a JEM-1400Plus (JEOL Ltd., Tokyo, Japan) operated at an accelerating voltage of 100 kV. Images were acquired using a CCD camera (EM-14830RUBY2; JEOL Ltd.).

### 4.8. Histological Analysis

After applying SE or IM to the three-dimensional human epidermis for 8 days, the tissue was fixed with 4% paraformaldehyde buffer (pH 7.4) at 4 °C overnight. The paraffin-embedded tissue specimens were sectioned at a thickness of approximately 3 µm and stained with hematoxylin and eosin (H&E), in accordance with standard protocols. Histological images were acquired using an Axio Imager M2 microscope (Carl Zeiss, Oberkochen, Germany).

### 4.9. Statistical Analysis

All data are expressed as mean ± standard deviation (SD). Statistical comparisons among three or more groups were performed using either Dunnett’s or Tukey’s post hoc test, as appropriate, with JMP^®^ Pro software (version 17.2.0; SAS Institute Inc., Cary, NC, USA).

## 5. Conclusions

In this study, we identified SE as a multimineral preparation that modulates key molecular pathways governing intercellular adhesion and proteolytic balance in a three-dimensional human epidermal model. SE promoted a gene expression profile consistent with reinforced corneodesmosomal integrity and a more controlled protease–inhibitor environment, changes that were accompanied by morphological features indicative of enhanced epidermal maturation. Collectively, these findings suggest that SE contributes to the maintenance of epidermal barrier homeostasis through the coordinated regulation of corneodesmosome turnover and protease activity. SE therefore represents a promising natural ingredient that improves skin barrier functions, particularly in dry or sensitive skin conditions. This study had several limitations. The spatial distribution of corneodesmosomal proteins was not evaluated, preventing conclusions about their localization within differentiating epidermal layers. Moreover, all experiments were performed in an in vitro model, and the physiological relevance of SE has yet to be verified in vivo. Future studies incorporating protein localization analyses and in vivo validation will be essential to substantiate the barrier-modulating effects of SE and clarify its potential applications in skin care or dermatological interventions.

## Data Availability

The original contributions presented in this study are included in the article/Supplementary Material.

## Supplementary Materials

The following supporting information can be downloaded at: https://www.mdpi.com/article/10.3390/molecules30234592/s1, Supplementary Figure S1. Full-length, uncropped western blot images; Supplementary Table S1. PCR primer sequences.
