# Supplementary material for: Dual Regulation of Corneodesmosome Formation by Shotokuseki Extract Enhances Skin Barrier Homeostasis
Source: Molecules. 2025 Nov 29;30(23):4592. doi: 10.3390/molecules30234592 (PMC12693148; doi:10.3390/molecules30234592)
Supplement: Supplementary file 1 [file molecules-30-04592-s001.zip › Supplementary figure S1.pdf]

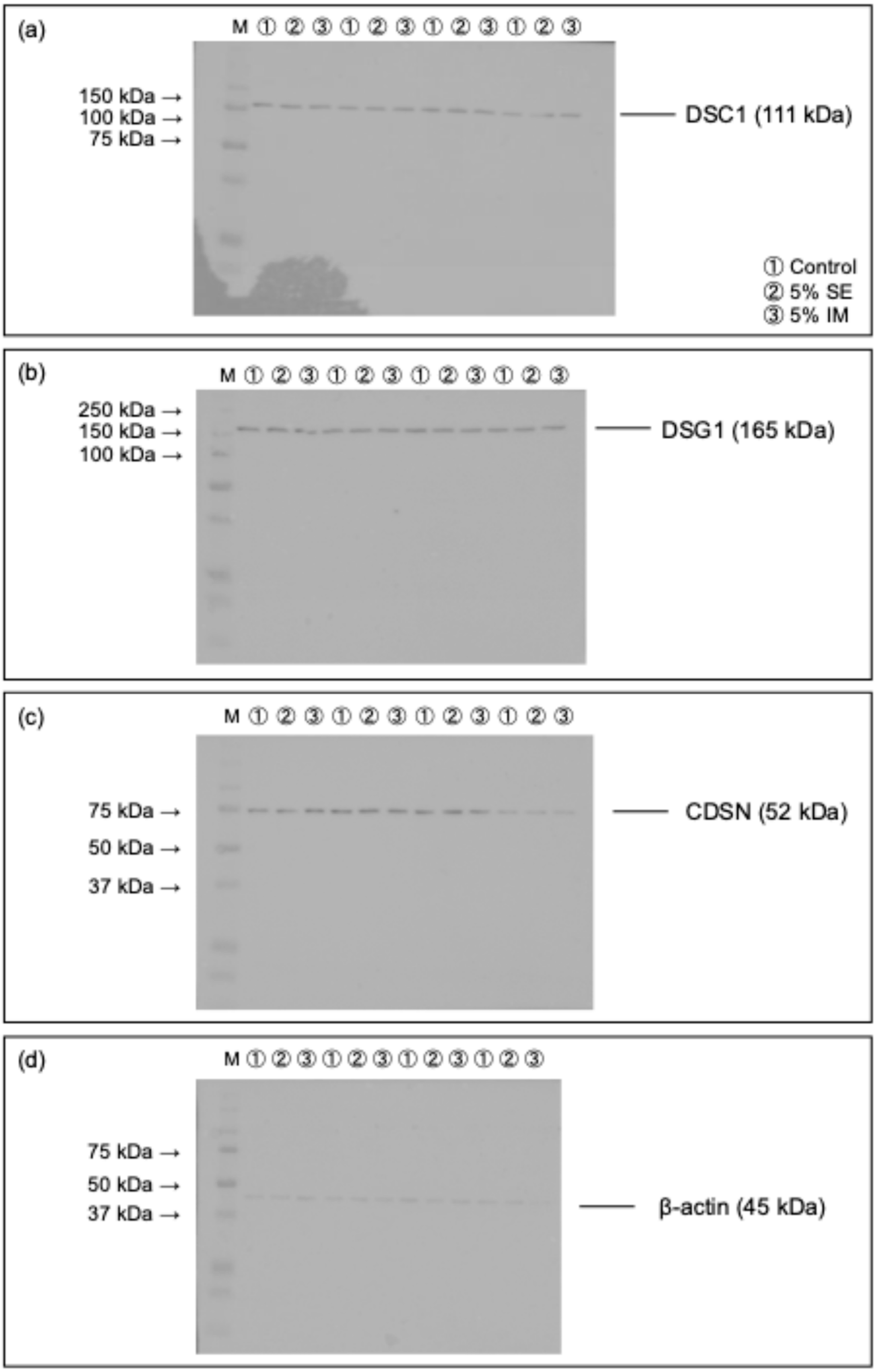

Supplementary figure S1. Full-length, uncropped western blot images. Overlay of chemiluminescence image and digitized image. (a) DSC1, (b) DSG1, and (c) CDSN expression were normalized by (d) β-actin.
