# Supplementary material for: Dual Regulation of Corneodesmosome Formation by Shotokuseki Extract Enhances Skin Barrier Homeostasis
Source: Molecules. 2025 Nov 29;30(23):4592. doi: 10.3390/molecules30234592 (PMC12693148; doi:10.3390/molecules30234592)
Supplement: Supplementary file 1 [file molecules-30-04592-s001.zip › Supplementary Table S1.pdf]

Supplementary Table S1. PCR primer sequences.

| Primer | | Sequence |
|---|---|---|
| *DSC1* | Forward | 5’-CAGGCTGAAACACTTGTAGGC-3’ |
| | Reverse | 5’-TAGGCTGGCCGACTTGAG-3’ |
| *DSG1* | Forward | 5’-AGATGCCTGACTTGCGAGAT-3’ |
| | Reverse | 5’-CTATCATGCCGGAAGTTGGT-3’ |
| *DSG3* | Forward | 5’-TTCCTGATCACATGTCGGGC-3’ |
| | Reverse | 5’-CACCAGTGAGTTTGAGGCACT-3’ |
| *CDSN* | Forward | 5’-CCAATCCAGTGGCAAAATCA-3’ |
| | Reverse | 5’-CATGCAAGGGTGACCAGAAGA-3’ |
| *KLK5* | Forward | 5’-CACAAGGGTAATCTCCCCAG-3’ |
| | Reverse | 5’-AGA TGACACCA TGTTCTGCG-3’ |
| *KLK7* | Forward | 5’-GGGTACCTCTGCACACCAAC-3’ |
| | Reverse | 5’-GGATGTCAAGCTCATCTCCC-3’ |
| *SPINK5* | Forward | 5’-AGCCCCCAGTCTGTATCCTT-3’ |
| | Reverse | 5’-CTCCCTTTGCAGAACTCAGG-3’ |
| *SLPI* | Forward | 5’-AGCGTGACTTGAAGTGTTGCATG-3’ |
| | Reverse | 5’-GAAAGGACCTGGACCACACAGA-3’ |
| *PI3* | Forward | 5’-CAGTCTCCACTAAGCCTGGC-3’ |
| | Reverse | 5’-GTGGATGAGAGAGGCAGCTC-3’ |
